# An Economic Model of Gambling Behaviour: A Two-Stage Approach

**DOI:** 10.1007/s10899-022-10146-2

**Published:** 2022-07-22

**Authors:** Lachlan Cameron, Jemimah Ride, Nancy Devlin

**Affiliations:** Health Economics Unit, School of Population and Global Health, The University of Melbourne, 207 Bouverie St, Carlton, VIC 3053 Australia

**Keywords:** Gambling participation, Gambling-related harm, Addiction, Intervention, Decision making, Cognitive bias

## Abstract

Gambling can cause significant harms and these can result in a net negative utility from participation, although lower levels of participation have potential benefits and can yield positive net utility. It is therefore important to understand and distinguish between these two stages of gambling behaviour. Currently, economic models have had limited focus on explaining why someone would gamble despite it yielding a negative utility. Here, we present a two-stage model, motivated by empirical literature and intuitive assumptions, that improves on existing economic models by distinguishing between the likelihood of gambling participation and of gambling that yields a negative utility. The model’s predictions are empirically testable, consistent with existing literature, and add new insights. The model’s ability to distinguish between the two stages helps to inform interventions that aim to reduce the prevalence of gambling-related harm while avoiding the need for restrictive approaches that aim to eliminate gambling altogether.

## Introduction

Gambling can cause harms such as financial difficulties, emotional distress, decreased work performance, relationship stress, poor physical health, and criminal activity (Browne et al., 2017), and has negative externalities to society (Goodwin et al., 2017). Worldwide, estimates for the prevalence of people with significant gambling-related harms vary between 0.12 and 5.8% of the legal-aged population (Calado & Griffiths, 2016)^1^. Despite the potential harm, gambling has become normalized as a legal recreational activity in many countries (Livingstone & Rintoul, 2020). There is evidence that low levels of gambling can improve life satisfaction (Humphreys et al., 2021). Therefore, the policy goal is usually not to prevent gambling entirely but to implement interventions that limit gambling-related harms.

Utility, a latent construct similar to welfare, satisfaction, and pleasure, is a term regularly used in economics to explain and predict behaviour (Kapteyn, 1985). People are understood as aiming to maximise their utility, and so are expected to choose the course of action that they expect to yield the higher utility. With this in mind, economists have struggled to explain gambling participation for two reasons. First, because economic theory generally assumes individuals have diminishing returns to wealth, and hence risk averse preferences over wealth (Nyman et al., 2008). Second, gambling usually has an expected monetary loss (Levitt, 2004; Churchill & Farrell, 2018). These indicate that an individual would have a higher expected utility by keeping a certain amount instead of gambling it, and hence would not participate in gambling if acting rationally. Attempts by economists to explain gambling participation include postulating that utility from a monetary reward through gambling is higher than utility from earning an equivalent amount of money by working (Nyman et al., 2008, 2013), market imperfection that results in gamblers overestimating the probability of winning (Gong & Zhu, 2019), and psychological benefits of gambling that have a positive effect on utility (Conlisk, 1993; Churchill & Farrell, 2018). However, these explanations maintain the notion that people will only gamble if it gives them a positive utility. This is problematic because a result of gambling-related harms is that gambling may give an individual a negative utility, and hence the existing models have limited focus on explaining why someone might gamble despite it resulting in significant harms for the individual. Churchill and Farrell (2018) describe gambling that yields a negative utility as being due to gambling addiction, but do not model how someone might develop this gambling behaviour.

The aim of this paper is to address this gap in the literature by developing an economic model that explains both (1) gambling participation and (2) the development of gambling that yields a negative utility. It describes these as the two stages of gambling behaviour, where someone who is more likely to participate in gambling is not necessarily more likely to develop gambling that yields a negative utility. Distinguishing between the two stages of gambling allows the model to help interventions limit gambling-related harm while avoiding the need for restrictive approaches that eliminate gambling altogether. The model explains (1) the factors that affect the likelihood of gambling participation, (2) how gambling that yields a negative utility develops from initial participation, including the factors that affect the likelihood of this, and (3) why someone may continue to gamble despite it yielding a negative utility. It is also consistent with the suggestion in Gong and Zhu (2019), that market imperfections may affect gambling behaviour, by incorporating cognitive biases.

Section “The Model” introduces the model, discussing the inspirations, assumptions, factors determining the inputs, and how it incorporates cognitive biases. Section “Two Stages of Gambling Behaviour” then illustrates how the model explains and distinguishes between the two stages, discussing the factors that affect (1) the likelihood of participating in gambling and (2) the likelihood of developing gambling that yields a negative utility. Finally, “Conclusion” section discusses the model’s predictions, provides some concluding remarks, and suggests avenues for future research.

## The Model

This model uses environmental and personal factors to explain the two stages underpinning gambling behaviour, allowing that people who are more likely to participate in gambling are not necessarily more likely to develop gambling that yields a negative utility. To do this effectively, it must address three points. First, it must explain why people participate in gambling despite having diminishing returns to wealth and gambles having an expected monetary loss. Second, it must explain how gambling that yields a negative utility develops from initial participation, where the factors that affect this at least partially differ from the factors that affect the likelihood of participation so that a distinction between the two stages can be made. Third, it must explain why someone would continue gambling despite it yielding a negative utility.

The model combines a baseline expected utility of gambling with a term for unsatisfied gambling cravings influenced by past gambling behaviour. In brief, the model can be described as

$${\text{Utility}}\;{\text{of}}\;{\text{Gambling}}\; = \;{\text{Time}}\;{\text{Discounted}}\;{\text{Expected}}\;{\text{Utility}}\;{\text{of}}\;{\text{Current}}\;{\text{Gamble}} - {\text{Unsatisfied}}\;{\text{Gambling}}\;{\text{Cravings}}$$

### Time Discounted Expected Utility of Current Gamble

Inspired by Conlisk (1993) and Churchill and Farrell (2018), the ‘time discounted expected utility of current gamble’ consists of (1) the psychological effects of gambling and (2) the monetary aspect of gambling.

The utility is time discounted because some of the psychological effects of a current gamble are experienced in future time periods. The future impact on utility is an expectation because of the uncertain nature of gambling and the dependence of the monetary and psychological effects on the result of the gamble. For example, the monetary utility will be positive if the gamble yields a profit and negative if it yields a loss. The respective values of the utility for a profit and a loss and the probability of each outcome determine the expected monetary utility. Similarly, the psychological effects experienced will differ depending on the result of the gamble, more for a loss. The model treats a gambling session, comprised of multiple individual bets, as a single unit of time. For simplicity, we assume that the outcome of a gambling session is equal to the expected outcome.

#### Psychological Effects of Gambling

Psychological effects of gambling can be positive and/ or negative (Churchill & Farrell, 2018). If the total positive psychological effects are greater than the total negative psychological effects then the net psychological effect of gambling is positive, and hence the utility associated with the psychological effects is positive.

Intuitively, excessive gambling leads to net negative psychological effects. Therefore, although the net psychological effects can be positive for some amounts gambled, we assume that, for each individual, gambling more than a certain amount^2^ results in the utility associated with psychological effects contributing negatively to total utility.

Let $A_{it}$ be defined as the amount individual *i* gambles in a gambling session in period *t*. To capture the above, the utility associated with the psychological effects of gambling $A_{it}$ in period *t*, represented by $U^P_{it}(A_{it})$, must be such that $\frac{d^2 U^P_{it}(A_{it})}{d A_{it}^2} < 0$, but where $U^P_{it}(A_{it})$ and $\frac{dU^P_{it}(A_{it})}{dA_{it}}$ can be positive for some amounts gambled.

Consistent with this, let $U^P_{it}(A_{it})$ be given by

1 $$\begin{aligned} U_{it}^P(A_{it}) = \alpha _{Pi} A_{it} + \frac{\alpha _{PPi}}{2}A_{it}^2 \end{aligned}$$

where $\alpha _{Pi}$ and $\alpha _{PPi}$ are defined as single terms capturing the factors that determine the utility associated with the psychological effects of gambling, with $\alpha _{Pi} \in \mathbb {R}$ and $\alpha _{PPi}<0$. If $\alpha _{Pi} > 0$ then the individual has net positive psychological effects for some amounts gambled, and $\alpha _{Pi} < 0$ implies the individual has net negative psychological effects for all amounts gambled.

Positive psychological effects include excitement, leisure or fun, socialising, and hope of winning big money (Francis et al., 2015). Negative psychological effects include feelings of guilt, relationship and financial stress, and anxiety (Browne et al., 2016). In the remainder of this paper, the net effect of these positive and negative emotions is referred to as the ‘net pleasure from gambling’, where large positive emotions relative to negative emotions means the individual has greater net pleasure from gambling and hence will have higher^3^ values of $\alpha _{Pi}$ and $\alpha _{PPi}$.

Negative psychological effects also derive from the time and effort required for gambling. Easier access to gambling, depending in turn on factors such as proximity to gambling venues and the availability of online gambling, hence results in higher values of $\alpha _{Pi}$ and $\alpha _{PPi}$.

Some of these psychological effects would be experienced during the gambling session, and others after the gambling session. In general, the positive emotions from gambling occur during the gambling session and the negative emotions are longer term effects. Therefore, we assume the net pleasure during the gambling session is greater than the net pleasure experienced after the gambling session. This implies that the expected utility associated with the psychological effects is greater for people who discount the future more heavily, and hence a greater time discount rate results in higher values of $\alpha _{Pi}$ and $\alpha _{PPi}$.

While $\alpha _{Pi}$ and $\alpha _{PPi}$ are positively correlated, $\alpha _{PPi}$ captures factors that are more sensitive to the amount gambled rather than participation alone. Therefore, someone with a higher value of $\alpha _{PPi}$, for a given value of $\alpha _{Pi}$, is someone whose negative aspects of net pleasure from gambling are less sensitive to the extra risks associated with larger amounts gambled or stems from a gambling environment that requires less effort to gamble larger amounts.

#### Monetary Aspect of Gambling

Consistent with risk aversion over wealth, we assume that the expected utility of the monetary aspect of gambling is negative for any amount gambled and becomes more negative at an increasing rate as the amount gambled increases.

To capture this, the utility of the monetary aspect when gambling $A_{it}$ in period *t*, represented by $U^M_{it}(A_{it})$, must be such that $U^M_{it}(A_{it}), \frac{dU^M_{it}(A_{it})}{dA_{it}}, \frac{d^2 U^M_{it}(A_{it})}{dA_{it}^2} < 0$

Consistent with this, let $U^M_{it}(A_{it})$ be given by

2 $$\begin{aligned} U_{it}^M(A_{it}) = \alpha _{Mi} A_{it} + \frac{\alpha _{MMi}}{2}A_{it}^2 \end{aligned}$$

where $\alpha _{Mi}$ and $\alpha _{MMi}$ are defined as single terms capturing the factors that determine the utility associated with the monetary aspect of gambling, with $\alpha _{Mi},\alpha _{MMi} < 0$. Greater levels of risk aversion^4^ are reflected in lower values of $\alpha _{Mi}$ and $\alpha _{MMi}$.

These negative values of $\alpha _{Mi}$ and $\alpha _{MMi}$ also reflect the expected monetary loss resulting from the odds offered by gambling vendors which are designed to make a profit for the vendor^5^ (Levitt, 2004). This element of the monetary aspect of gambling is susceptible to cognitive biases that can cause people to overestimate their probability of winning, resulting in a biased expected utility.

#### Time Discounted Expected Utility of Current Gamble

The sum of $U^P_{it}(A_{it})$ and $U^M_{it}(A_{it})$ is defined as the ‘time discounted expected utility of current gamble’. By combining (1) and (2), it is given by

3 $$\begin{aligned} (\alpha _{Pi} + \alpha _{Mi}) A_{it} + \frac{\alpha _{PPi} + \alpha _{MMi}}{2}A_{it}^2 \end{aligned}$$

This is the total expected utility of gambling in the absence of cravings. Hence, this acts as a baseline expected utility that determines the gambling behaviour of a non-gambler (someone who has not previously gambled, and hence has no cravings).

### Unsatisfied Gambling Cravings

If someone participates in gambling they become a current gambler. They will remain a current gambler unless they abstain from gambling (in which period they will become an ex-gambler). In the model, a current gambler differs from a non-gambler because they have cravings to gamble that are caused by past gambling. This relates to the ‘unsatisfied gambling cravings’ part of the utility function, which is inspired by Becker’s Theory of Rational Addiction (Becker & Murphy, 1988), and Gruber’s extension (Gruber & Köszegi, 2001), that model adjacent complementarity of addictive goods.

There are six intuitive assumptions about how gambling cravings behave in this model.

#### **Assumption 1**

The level of craving has a positive relationship with the amount previously gambled, the length of gambling history, and individual propensity to crave gambling.

#### **Assumption 2**

The marginal utility of gambling in the current period is larger, all else equal, when the level of craving is larger.

#### **Assumption 3**

If not satisfied, cravings have a negative effect on utility, with a larger effect the greater the level of unsatisfied cravings. Gambling a non-zero amount at least partially satisfies these cravings. A greater level of craving implies that a larger amount must be gambled to fully satisfy cravings. Satisfaction of cravings by gambling can only ever reduce the negative utility of unsatisfied cravings and cannot produce a positive utility.

#### **Assumption 4**

All else equal, unsatisfied cravings cause a greater utility loss for people with a greater baseline utility of gambling.

#### **Assumption 5**

If a current gambler abstains from gambling, their cravings persist but diminish over time. This implies that cravings affect ex-gamblers as well as current gamblers.

#### **Assumption 6**

People do not consciously consider cravings when making their decision to gamble (or they are unaware that their current gambling decision will affect their future cravings). For a non-gambler, this means they do not consider that they will have any cravings if they gamble. For a current gambler, this means they do not consider that their cravings will increase if they continue gambling, or decrease if they sufficiently reduce their amount gambled. For an ex-gambler, this means they do not consider that their cravings will further diminish if they continue abstaining, or increase if they relapse on their abstinence. This assumption implies that, although current gambling affects future cravings, people do not factor future cravings into their current decision to gamble.

$S_{it}$ is defined as the stock of past gambling, where $S_{it} \ge 0$. As in Becker and Murphy (1988)^6^, the evolution of the stock of past gambling is given by

4 $$\begin{aligned} S_{it} = (1-d_i)S_{i(t-1)} + A_{i(t-1)} \end{aligned}$$

where $d_i$ denotes the ‘depreciation’ of existing stock, with $d_i \in (0,1)$. In gambling terms, $d_i$ determines the extent to which cravings persist. A lower value of $d_i$ results in both a faster buildup of cravings and cravings that diminish more slowly when abstaining from gambling. Including a stock of past gambling, as opposed to having cravings dependent on the most recent period of gambling only, implies that a longer history of gambling, as well as a greater amount gambled, increases cravings, which is necessary to satisfy Assumption 1. It also implies that cravings persist (although diminish) if the individual abstains, which satisfies Assumption 5.

$\alpha _{Ci}$ is defined as a single term capturing the factors that determine individual propensity to crave gambling, with $\alpha _{Ci} > 0$. ‘$\alpha _{Ci} S_{it}$’ is defined as the level of gambling cravings.

To satisfy all assumptions, the utility associated with unsatisfied cravings, represented by $U_{it}^C(A_{it},S_{it})$^7^, must be such that $U_{it}^C(A_{it},S_{it}) \ge 0$ and $\frac{\partial U_{it}^C(A_{it},S_{it})}{\partial A_{it}} \le 0$ (Assumption 3), $\frac{\partial U_{it}^C(A_{it},S_{it})}{\partial (\alpha _{Ci} S_{it})} > 0$ (Assumption 1), $\frac{\partial ^2 U_{it}^C(A_{it},S_{it})}{\partial A_{it} \partial (\alpha _{Ci} S_{it})} < 0$ (Assumption 2), and $\frac{\partial U_{it}^C(A_{it},S_{it})}{\partial \alpha _{Pi}},\frac{\partial U_{it}^C(A_{it},S_{it})}{\partial \alpha _{PPi}},\frac{\partial U_{it}^C(A_{it},S_{it})}{\partial \alpha _{Mi}},\frac{\partial U_{it}^C(A_{it},S_{it})}{\partial \alpha _{MMi}} > 0$ (Assumpion 4).

For simplicity, $U_{it}^C(A_{it},S_{it})$ is constructed such that cravings are exactly satisfied when individuals are maximising their utility^8^. Therefore, and to satisfy the six assumptions, let $U_{it}^C(A_{it},S_{it})$ be given by

5 $$\begin{aligned} U_{it}^C(A_{it},S_{it}) = \max \left\{ \alpha _{Ci} S_{it}\left[ \frac{-(\alpha _{Pi} + \alpha _{Mi} + \alpha _{Ci} S_{it})}{\alpha _{PPi} + \alpha _{MMi}} - A_{it} \right] ,0\right\} \end{aligned}$$

If $A_{it} < \frac{-(\alpha _{Pi} + \alpha _{Mi} + \alpha _{Ci} S_{it})}{\alpha _{PPi} + \alpha _{MMi}}$ then the individual is gambling an insufficient amount to fully satisfy cravings, which implies (5) is positive and hence a negative effect on utility caused by unsatisfied cravings. The max function restricts (5) to a non-negative value to prevent cravings from having a positive effect on total utility.

Based on existing literature, the factors influencing an individual’s propensity to crave gambling include their propensity to gamble for mood moderation, propensity to chase losses, and susceptibility and exposure to gambling advertising.

A Gambling Craving Scale was developed by Young and Wohl (2009). They postulate that cravings to gamble are comprised of (1) anticipation of enjoyment from gambling, (2) a strong desire to gamble, and (3) an expectation that gambling would relieve negative affect. (1) and (3) are consistent with the notion in Blaszczynski’s Pathways Model (Blaszczynski & Nower, 2002) that one reason people continue gambling, and increase their gambling level, is to re-live emotions previously experienced during gambling to moderate their mood. This is supported in a qualitative study by Wood and Griffiths (2007), which found that some individuals gamble for an escape from, or to calm, their hyperactive mood, or for excitement to boost their low mood, and that gambling for mood moderation contributed to cravings to gamble.

The notion that loss chasing contributes to cravings is also consistent with Blaszczynski’s Pathways Model, and is supported by empirical evidence (Ciccarelli et al., 2019; Young & Wohl, 2009).

Gambling advertising has been found to trigger gambling cravings (Binde, 2009; Hing et al., 2014).

Greater need for mood moderation, loss chasing, and susceptibility and exposure to gambling advertising result in an overall greater propensity to crave gambling, and hence a higher value of $\alpha _{Ci}$.

### The Total Expected Utility from Gambling

By combing Eqs. (3) and (5), the total expected utility for an individual from gambling $A_{it}$, when their stock of past gambling is $S_{it}$, in period *t*, is given by

6 $$\begin{aligned}&U_{it}(A_{it},S_{it}) = (\alpha _{Pi} + \alpha _{Mi})A_{it} + \frac{\alpha _{PPi} + \alpha _{MMi}}{2}A_{it}^2 \nonumber \\& \quad -\max \left\{ \alpha _{Ci} S_{it}\left[ \frac{-(\alpha _{Pi} + \alpha _{Mi} + \alpha _{Ci} S_{it})}{\alpha _{PPi} + \alpha _{MMi}} - A_{it} \right] ,0\right\} \end{aligned}$$

Note that (6) is not monotonically increasing in $A_{it}$. Therefore, maximising the utility of gambling in each period has a solution without introducing any constraints. However, we also assume that a decision to gamble is subject to available income.

Equation (6) represents the total expected utility of the current period’s gamble only. An individual’s discounted lifetime utility is found by summing (6) across all periods of the individual’s lifetime where utility in future periods is discounted by the individual’s time discount rate, $\delta _i$, with $\delta _i \in [0,1]$. Therefore, an individual’s discounted lifetime utility of gambling is given by

7 $$\begin{aligned} \sum _{t=0}^n (1 - \delta _i)^t U_{it}(A_{it},S_{it}) \end{aligned}$$

where *t* represents the period, with $t=0$ as the current period, and *n* is the number of remaining periods in the individual’s lifetime.

We postulate that it is the individual’s discounted lifetime utility that determines their well-being, but that, since they do not consciously consider cravings, they will aim to maximise utility in the current period only.

### Cognitive Biases

Cognitive biases can cause people to overestimate their probability of winning a gamble. Sources of such biases include gambler’s fallacy, overconfidence, inherent memory bias, and illusion of control (Fortune & Goodie, 2012). In some cases, an individual could believe that they have an expected monetary gain, despite an actual expected loss, and choose to gamble for this reason. This is consistent with the notion in Gong and Zhu (2019) that market imperfections are a reason that people gamble.

Cognitive biases could also lead to people overestimating positive psychological effects from gambling, such as the excitement or joy they will feel from a win, or underestimating future negative psychological effects of a current gamble, such as underestimating how guilty they will feel afterwards or the stress that gambling can cause. Overestimating the probability of winning may also create biases in relation to psychological effects, since it will cause people to overestimate the likelihood of feeling the positive psychological effects more associated with winning.

Importantly, these biases will only affect the individual’s subjective belief about their probability of winning or emotions caused by gambling, but not the actual probability or emotions felt. This introduces the concept of a wedge between decision utility, the utility that decision makers aim to maximise, and experienced utility, the true pleasure and pain experienced as a result of the decision made, noted by Kahneman et al. (1997).

The model can represent either decision or experienced utility. Decision utility is given by (6) when the variables represent the individual’s subjective beliefs (e.g. their belief of the probability of winning, their expectations about future psychological effects of current gambling). Experienced utility is the objective prediction of (6) (determined by the actual probability of winning, the future psychological effects the individual will actually experience). The exception to this is that, since cravings to gamble are entirely internal, we assume that the variables in (5) are always associated with subjective beliefs.

To differentiate between the two sets of variables, the variables with *S* superscripts represent subjective beliefs, and *O* superscripts represent objective predictions. $\alpha _{Ci}$ and $d_i$ do not have superscripts because they are not susceptible to bias.

Distinguishing between the two sets of variables, the expected decision utility of gambling ($U_{it}^S$) is given by

8 $$\begin{aligned}&U_{it}^S(A_{it},S_{it}) = (\alpha _{Pi}^S + \alpha _{Mi}^S)A_{it} + \frac{\alpha _{PPi}^S + \alpha _{MMi}^S}{2}A_{it}^2 \nonumber \\& \quad -\max \left\{ \alpha _{Ci} S_{it}\left[ \frac{-(\alpha _{Pi}^S + \alpha _{Mi}^S + \alpha _{Ci} S_{it})}{\alpha _{PPi}^S + \alpha _{MMi}^S} - A_{it} \right] ,0\right\} \end{aligned}$$

and the expected experienced utility of gambling ($U_{it}^O$) is given by

9 $$\begin{aligned}&U_{it}^O(A_{it},S_{it}) = (\alpha _{Pi}^O + \alpha _{Mi}^O)A_{it} + \frac{\alpha _{PPi}^O + \alpha _{MMi}^O}{2}A_{it}^2 \nonumber \\& \quad -\max \left\{ \alpha _{Ci} S_{it}\left[ \frac{-(\alpha _{Pi}^S + \alpha _{Mi}^S + \alpha _{Ci} S_{it})}{\alpha _{PPi}^S + \alpha _{MMi}^S} - A_{it} \right] ,0\right\} \end{aligned}$$

If an individual overestimates their probability of winning then $\alpha _{Mi}^S > \alpha _{Mi}^O$ and $\alpha _{MMi}^S > \alpha _{MMi}^O$, and if they overestimate the net psychological effect of gambling then $\alpha _{Pi}^S > \alpha _{Pi}^O$ and $\alpha _{PPi}^S > \alpha _{PPi}^O$. Each of these biases will create a wedge between the two utilities such that the expected decision utility of gambling is greater than the expected experienced utility of gambling.

We maintain the assumption of an objective expected monetary loss, and hence have the restriction $\alpha _{Mi}^O < 0$. However, we relax the restriction on the subjective value to allow for the belief of an expected monetary gain, hence $\alpha _{Mi}^S \in \mathbb {R}$. We assume that the subjective psychological effect is still negative for people with cognitive biases if a sufficiently large amount is gambled^9^, and hence $\alpha _{PPi}^S < 0$, and to preserve risk-aversion over wealth we keep the restriction $\alpha _{MMi}^S < 0$.

Kahneman and Sugden (2005) postulate that, given individuals are likely to make errors about future utility, maximising experienced utility may be preferable to maximising decision utility. Consistent with this, we assume that individuals make their gambling decision to maximise their expected decision utility based on subjective beliefs, but that their well-being depends on their experienced utility.

## Two Stages of Gambling Behaviour

### Stage 1: Participation in Gambling

There are two key assumptions when using the model to predict the gambling behaviour of a utility maximising individual: they gamble to maximise their (1) expected decision utility and (2) utility in the current period. Therefore, the model predicts that the individual will only participate in gambling if there exists an amount $A_{it} > 0$ that yields a positive utility given by (8). This is true if and only if the amount gambled that maximises (8) is $A_{it} > 0$.

The amount gambled that maximises (8) is found by setting $\frac{\partial U_{it}^S}{\partial A_{it}}=0$. This yields

10 $$\begin{aligned} A_{it}(S_{it}) = \frac{-(\alpha _{Pi}^S + \alpha _{Mi}^S + \alpha _{Ci} S_{it})}{\alpha _{PPi}^S + \alpha _{MMi}^S} \end{aligned}$$

with a corner solution at 0 if $A_{it} < 0$ (indicating the individual will not gamble).

Since $\alpha _{PPi}^S,\alpha _{MMi}^S < 0$, (10) implies that a non-gambler ($S_{it} = 0$) will only participate in gambling if $\alpha _{Pi}^S > - \alpha _{Mi}^S$. This can occur for two reasons. First, if their expected psychological benefit from gambling is sufficiently positive to outweigh the expected utility loss from the monetary aspect of the gamble. Second, if the individual has cognitive biases that cause them to believe they have an expected monetary gain (i.e. $\alpha _{Mi}^S > 0$). Even if $\alpha _{Mi}^S$ is less than zero, cognitive biases still increase the likelihood that $\alpha _{Pi}^S > - \alpha _{Mi}^S$ (either through higher $\alpha _{Mi}^S$ or higher $\alpha _{Pi}^S$).

An individual will not definitely gamble if $A_{it} > 0$. As mentioned earlier, a decision to gamble is subject to available income, implying that people with lower incomes and who spend more money on substitute activities will be less likely to participate in gambling.

### Gambling Behaviour Over Time

If an individual participates in gambling, they will build up a stock of past gambling ($S_{it} > 0$), and hence have cravings to gamble. All else equal, this will cause the amount gambled given by (10) to increase. Note that gambling (10) is assumed to exactly satisfy cravings,^10^ leaving no negative utility associated with unsatisfied cravings. However, cravings still influence the utility of gambling by causing the amount gambled to increase over time. This increase in amount gambled will increase the stock of past gambling, and hence increase cravings further in the next period, causing another increase in amount gambled. This process continues, with the possibility of reaching an equilibrium level.

An equilibrium level of gambling is reached if $A_{it}$ converges to a point such that $A_{it} = A_{i(t-1)} = \bar{A_i}$, which will occur if and only if $S_{it}$ converges to a point such that $S_{it} = S_{i(t-1)} = \bar{S_i}$, where $\bar{S_i}$ and $\bar{A_i}$ denote the equilibrium levels of stock of past gambling and amount gambled respectively.

Individuals with $-(\alpha _{PPi}^S + \alpha _{MMi}^S) > \frac{\alpha _{Ci}}{d_i}$ will reach an equilibrium. At this equilibrium, the amount gambled is given by (see Appendix 1.1)

11 $$\begin{aligned} \bar{A_i} = \frac{-(\alpha _{Pi}^S + \alpha _{Mi}^S)}{\alpha _{PPi}^S + \alpha _{MMi}^S + \frac{\alpha _{Ci}}{d_i}} \end{aligned}$$

For individuals with $-(\alpha _{PPi}^S + \alpha _{MMi}^S) < \frac{\alpha _{Ci}}{d_i}$, the amount gambled will increase indefinitely over time.

If the individual does not have the available income to gamble the amount given by (10) then the model suggests they will gamble their total available income and, since their cravings will not be satisfied, will also have unsatisfied cravings.

### Stage 2: Developing Gambling that Yields a Negative Utility

Common classifications of significant gambling-related harms include problem gambling—“gambling behaviour that creates negative consequences for the gambler, others in his or her social network, or for the community" (Ferris & Wynne, 2001)—and gambling disorder—“a persistent, recurrent pattern of gambling that is associated with substantial distress or impairment" (Association et al., 2013). In this paper we discuss significant gambling-related harms in terms of gambling that yields a negative experienced utility—that is, when an individual gambles despite the negative consequences outweighing any positive psychological effects. This differs from classifications such as problem gambling and gambling disorder which are based on behaviour and the experienced effects of that behaviour. Someone can experience harmful effects from gambling while also experiencing the positive utility impacts, so that the overall effects of gambling on utility is still positive. Utility captures the individual’s own experience and not negative externalities except where the individual internalises these (Kapteyn, 1985).

**Fig. 1:**
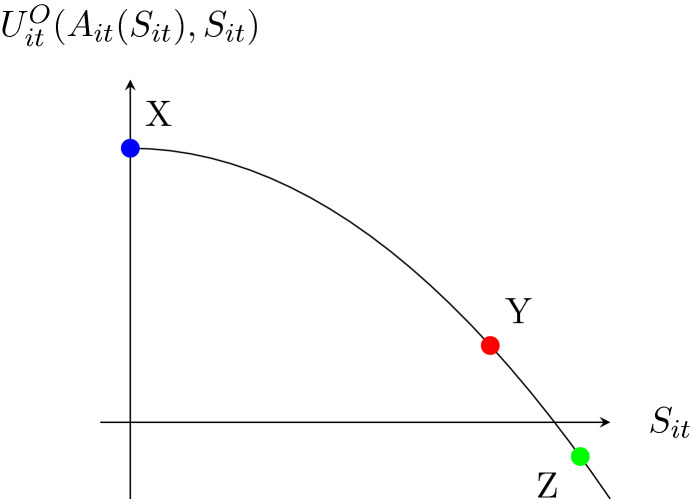
Indirect experienced utility when maximising decision utility in the current period. This is given by $(\alpha _{Pi}^O + \alpha _{Mi}^O)\left[ \frac{-(\alpha _{Pi}^S + \alpha _{Mi}^S + \alpha _{Ci} S_{it})}{\alpha _{PPi}^S + \alpha _{MMi}^S}\right] + \frac{\alpha _{PPi}^O + \alpha _{MMi}^O}{2}\left[ \frac{-(\alpha _{Pi}^S + \alpha _{Mi}^S + \alpha _{Ci} S_{it})}{\alpha _{PPi}^S + \alpha _{MMi}^S}\right] ^2$. Point X is positive (as shown here) if $\alpha _{Pi}^O > -\alpha _{Mi}^O$. If $\alpha _{Pi}^O < - \alpha _{Mi}^O$ then point X would be below the x-axis and gambling any positive amount would result in a negative experienced utility for all levels of stock

Figure 1 shows the indirect experienced utility, as a function of $S_{it}$, when gambling according to (10).^11^

A utility maximising individual who is currently a non-gambler ($S_{it} = 0$) will have a utility at point X in Fig.  1 in their first period of gambling. This gambling behaviour creates a stock of past gambling which will increase after each subsequent period of gambling, with the amount gambled in each period becoming larger as the stock increases, moving the individual along the curve down and to the right. If the individual’s stock of past gambling increases to a level such as at point Z then they will gamble an amount that yields a negative experienced utility. An individual at this point will continue gambling despite the negative utility because if they deviated from gambling according to (10) (e.g. abstained or gambled a smaller amount) they would have unsatisfied cravings which would result in a greater total utility loss. If the individual reaches an equilibrium level of stock such as at point Y then they will have a positive experienced utility from gambling in the long-run.

People who reach an equilibrium gambling amount will have a negative experienced utility from gambling in the long run if $(\alpha _{Pi}^O + \alpha _{Mi}^O)\bar{A_i} + \frac{\alpha _{PPi}^O + \alpha _{MMi}^O}{2}\bar{A_i}^2<0$. This will occur if (see Appendix 1.2)

12 $$\begin{aligned} 2\frac{\alpha _{Ci}}{d_i}> |\alpha _{PPi}^S + \alpha _{MMi}^S| - \left( \frac{(\alpha _{Pi}^S + \alpha _{Mi}^S)}{(\alpha _{Pi}^O + \alpha _{Mi}^O)}|\alpha _{PPi}^O + \alpha _{MMi}^O| - |\alpha _{PPi}^S + \alpha _{MMi}^S|\right) \end{aligned}$$

Therefore, a higher value of $\alpha _{Ci}$, lower value of $d_i$, higher values of $\alpha _{PPi}^S$ and $\alpha _{MMi}^S$, and cognitive biases^12^ increase the likelihood that this inequality holds, and hence the likelihood that the individual will develop gambling that yields a negative experienced utility.

An individual whose amount gambled increases indefinitely will develop gambling that yields a negative experienced utility. This also implies that a higher value of $\alpha _{Ci}$, lower value of $d_i$, and higher values of $\alpha _{PPi}^S$, and $\alpha _{MMi}^S$ (including cognitive biases) increase the likelihood of developing gambling that yields a negative experienced utility.

To summarise, the model makes the distinction between the two stages underpinning gambling behaviour through the inclusion of the cravings term and the structure of the terms for psychological effects and the monetary aspect. Most notably, people with greater propensity to crave gambling and/ or more persistent cravings (larger $\frac{\alpha _{Ci}}{d_i}$ ratio) are no more likely to participate in gambling, but do have a greater likelihood of developing gambling that yields a negative experienced utility from initial participation.

Secondly, $\alpha _{Pi}^S$ and $\alpha _{Mi}^S$ determine the likelihood of participating in gambling, and $\alpha _{PPi}^S$ and $\alpha _{MMi}^S$ affect the likelihood of developing gambling that yields a negative experienced utility. Although these terms are generally correlated (indicating some correlation between the respective likelihoods of participating in gambling and developing gambling that yields a negative experienced utility), the model suggests that people for whom the negative psychological effects are less sensitive to increases in amount gambled are more likely to develop gambling that yields a negative experienced utility but are no more likely to participate in gambling.

## Conclusion

Motivated by existing models, empirical literature, and intuitive assumptions, the model presented in this paper improves on existing economic models by making the distinction between the two stages underpinning gambling behaviour: gambling participation and the development of gambling that yields a negative utility.

Consistent with existing economic literature, the model postulates that individuals participate in gambling if their psychological effects of gambling are sufficiently positive to outweigh the negative impact of the monetary aspect (Churchill & Farrell, 2018) or if they have cognitive biases (Gong & Zhu, 2019). The model then distinguishes between the two stages by incorporating gambling cravings and relative sensitivity of psychological effects to the amount gambled, which affect the likelihood of developing gambling that yields a negative utility but not the initial decision to participate in gambling. The model explains continued participation despite having gambling that yields a negative utility by postulating that decreasing the amount gambled will give the individual unsatisfied cravings and result in a greater total utility loss.

This model provides a solid foundation which can be built upon further. Ways in which the model can be extended include (1) adding variability to the outcome of a gambling session, where different outcomes have different effects on future decision, (2) modelling individual bets within a gambling session, (3) individualizing the effect that each factor has on gambling, and (4) adding probability as a choice variable.

The model predicts that people who are less risk averse, have greater net pleasure from gambling, discount the future more heavily, and have easier access to gambling are more likely to participate in gambling, and that people who have greater propensity to crave gambling, more persistent cravings, negative aspects of net pleasure from gambling that are less sensitive to increases in amount gambled, and are subject to gambling environments more conducive to gambling large amounts are, all else equal, more likely to develop gambling that yields a negative utility from initial participation. Cognitive biases are a common factor which the model predicts will increase the likelihoods of both participation and gambling that yields a negative utility. These predictions are intuitive and empirically testable, and consistent with existing empirical literature^13^.

The model’s ability to distinguish between the two stages helps interventions target the sources of gambling-related harms to reduce their prevalence while avoiding the need for restrictive approaches that aim to eliminate gambling altogether. It suggests that interventions should aim to reduce gambling cravings and limit how conducive gambling environments are to gambling large amounts. Examples of such interventions include restricting gambling advertising, social promotion of less harmful activities which can moderate mood in similar ways to gambling, minimum time between bets, maximum bet sizes, deposit limits, and the removal of ATMs in and near gambling venues. This highlights the model’s important real-world applications in both predicting gambling behaviour and informing interventions.

## Data Availability

Not applicable.

## Appendix 1

### Equilibrium amount gambled

First, consider people with $\frac{\alpha _{Ci}}{d_i} < - (\alpha _{PPi}^S + \alpha _{MMi}^S)$.

The evolution of the stock of past gambling, when the individual is gambling (10), is given by

$$\begin{aligned} S_{t}&= (1-d) S_{i(t-1)} + A_{i(t-1)} \\&= (1-d) S_{i(t-1)} + \frac{-(\alpha _{Pi}^S + \alpha _{Mi}^S + \alpha _{Ci} S_{i(t-1)})}{\alpha _{PPi}^S +\alpha _{MMi}^S } \\&= \frac{((1-d)(\alpha _{PPi}^S +\alpha _{MMi}^S) - \alpha _{Ci}) }{\alpha _{PPi}^S +\alpha _{MMi}^S}S_{i(t-1)} - \frac{ (\alpha _{Pi}^S + \alpha _{Mi}^S )}{\alpha _{PPi}^S +\alpha _{MMi}^S } \end{aligned}$$

Therefore, the stock of past gambling will increase if

$$\begin{aligned}&\frac{((1-d)(\alpha _{PPi}^S +\alpha _{MMi}^S) - \alpha _{Ci}) }{\alpha _{PPi}^S +\alpha _{MMi}^S}S_{i(t-1)} - \frac{ (\alpha _{Pi}^S + \alpha _{Mi}^S )}{\alpha _{PPi}^S +\alpha _{MMi}^S }> S_{i(t-1)} \\ \implies&((1-d)(\alpha _{PPi}^S +\alpha _{MMi}^S) - \alpha _{Ci})S_{i(t-1)} - (\alpha _{Pi}^S + \alpha _{Mi}^S )< (\alpha _{PPi}^S +\alpha _{MMi}^S)S_{i(t-1)} \\ \implies&-(\alpha _{Pi}^S + \alpha _{Mi}^S)< ((\alpha _{PPi}^S +\alpha _{MMi}^S) - (1-d)(\alpha _{PPi}^S +\alpha _{MMi}^S) + \alpha _{Ci}) S_{i(t-1)} \\ \implies&-(\alpha _{Pi}^S + \alpha _{Mi}^S) < (d(\alpha _{PPi}^S +\alpha _{MMi}^S) + \alpha _{Ci}) S_{i(t-1)} \\ \implies&\frac{-(\alpha _{Pi}^S + \alpha _{Mi}^S)}{d(\alpha _{PPi}^S +\alpha _{MMi}^S) + \alpha _{Ci}} > S_{i(t-1)} \end{aligned}$$

where the last step is correct because $\frac{\alpha _{Ci}}{d_i} < - (\alpha _{PPi}^S + \alpha _{MMi}^S)$ implies $d(\alpha _{PPi}^S +\alpha _{MMi}^S) + \alpha _{Ci} < 0.$

Conversely, the stock of past gambling will decrease if $\frac{-(\alpha _{Pi}^S + \alpha _{Mi}^S)}{d(\alpha _{PPi}^S +\alpha _{MMi}^S) + \alpha _{Ci}} < S_{i(t-1)}$.

This implies that the stock of past gambling will converge (either from above or below) to $\bar{S_i} = \frac{-(\alpha _{Pi}^S + \alpha _{Mi}^S)}{d(\alpha _{PPi}^S +\alpha _{MMi}^S) + \alpha _{Ci}}$.

When the stock of past gambling is in equilibrium, the amount gambled must also be in equilibrium since it is the changing stock that affects the amount gambled across periods. Therefore, $A_{it} = \bar{A_i}$ when $S_{it} = S_{i(t-1)} = \bar{S_i}$, then from (4)

$$\begin{aligned}&\bar{S_i} = (1-d)\bar{S_i} + \bar{A_i} \\ \implies&d \bar{S_i} = \bar{A_i} \\ \implies&\bar{A_i} = \frac{-(\alpha _{Pi}^S + \alpha _{Mi}^S)}{(\alpha _{PPi}^S +\alpha _{MMi}^S) + \frac{\alpha _{Ci}}{d_i}} \end{aligned}$$

This completes the proof.

Note that for people with $\frac{\alpha _{Ci}}{d_i} > - (\alpha _{PPi}^S + \alpha _{MMi}^S)$, $d(\alpha _{PPi}^S +\alpha _{MMi}^S) + \alpha _{Ci} < 0$. Therefore, for these people, the stock of past gambling increases if

$$\begin{aligned} \frac{-(\alpha _{Pi}^S + \alpha _{Mi}^S)}{d(\alpha _{PPi}^S +\alpha _{MMi}^S) + \alpha _{Ci}} < S_{i(t-1)} \end{aligned}$$

which is always true (if $\alpha _{Pi}^S > - \alpha _{Mi}^S$) since $S_{i(t-1)} \ge 0$. This completes the proof that the amount gambled increases indefinitely for people with $\frac{\alpha _{Ci}}{d_i} > - (\alpha _{PPi}^S + \alpha _{MMi}^S)$.

### Long-run negative utility

Given (11),

$$\begin{aligned}&(\alpha _{Pi}^O + \alpha _{Mi}^O)\bar{A_i} + \frac{\alpha _{PPi}^O + \alpha _{MMi}^O}{2}\bar{A_i}^2< 0 \\ \implies&(\alpha _{Pi}^O + \alpha _{Mi}^O) + \frac{\alpha _{PPi}^O + \alpha _{MMi}^O}{2}\bar{A_i}< 0 \\ \implies&(\alpha _{Pi}^O + \alpha _{Mi}^O) + \frac{\alpha _{PPi}^O + \alpha _{MMi}^O }{2}\left[ \frac{-(\alpha _{Pi}^S + \alpha _{Mi}^S)}{\alpha _{PPi}^S + \alpha _{MMi}^S + \frac{\alpha _{Ci}}{d_i}}\right]< 0 \\ \implies&\frac{\alpha _{PPi}^O + \alpha _{MMi}^O }{2}\left[ \frac{-(\alpha _{Pi}^S + \alpha _{Mi}^S)}{\alpha _{PPi}^S + \alpha _{MMi}^S + \frac{\alpha _{Ci}}{d_i}}\right]< -(\alpha _{Pi}^O + \alpha _{Mi}^O) \\ \implies&-\frac{(\alpha _{Pi}^S + \alpha _{Mi}^S)(\alpha _{PPi}^O + \alpha _{MMi}^O)}{2}> -(\alpha _{PPi}^S + \alpha _{MMi}^S + \frac{\alpha _{Ci}}{d_i})(\alpha _{Pi}^O + \alpha _{Mi}^O) \\ \implies&\frac{-(\alpha _{Pi}^S + \alpha _{Mi}^S)(\alpha _{PPi}^O + \alpha _{MMi}^O)}{(\alpha _{Pi}^O + \alpha _{Mi}^O)}> -2(\alpha _{PPi}^S + \alpha _{MMi}^S) -2(\frac{\alpha _{Ci}}{d_i}) \\ \implies&\frac{-(\alpha _{Pi}^S + \alpha _{Mi}^S)(\alpha _{PPi}^O + \alpha _{MMi}^O)}{(\alpha _{Pi}^O + \alpha _{Mi}^O)} + 2(\alpha _{PPi}^S + \alpha _{MMi}^S)> -2\frac{\alpha _{Ci}}{d_i} \\ \implies&\frac{(\alpha _{Pi}^S + \alpha _{Mi}^S)(\alpha _{PPi}^O + \alpha _{MMi}^O)}{(\alpha _{Pi}^O + \alpha _{Mi}^O)} - 2(\alpha _{PPi}^S + \alpha _{MMi}^S)< 2\frac{\alpha _{Ci}}{d_i} \\ \implies&\frac{(\alpha _{Pi}^S + \alpha _{Mi}^S)(\alpha _{PPi}^O + \alpha _{MMi}^O)}{(\alpha _{Pi}^O + \alpha _{Mi}^O)} - (\alpha _{PPi}^S + \alpha _{MMi}^S) - (\alpha _{PPi}^S + \alpha _{MMi}^S)< 2\frac{\alpha _{Ci}}{d_i} \\ \implies&\frac{-(\alpha _{Pi}^S + \alpha _{Mi}^S)|\alpha _{PPi}^O + \alpha _{MMi}^O|}{(\alpha _{Pi}^O + \alpha _{Mi}^O)} + |\alpha _{PPi}^S + \alpha _{MMi}^S| + |\alpha _{PPi}^S + \alpha _{MMi}^S| < 2\frac{\alpha _{Ci}}{d_i} \\ \implies&2\frac{\alpha _{Ci}}{d_i}> |\alpha _{PPi}^S + \alpha _{MMi}^S| - \left[ \frac{(\alpha _{Pi}^S + \alpha _{Mi}^S)}{(\alpha _{Pi}^O + \alpha _{Mi}^O)}|\alpha _{PPi}^O + \alpha _{MMi}^O| - |\alpha _{PPi}^S + \alpha _{MMi}^S|\right] \end{aligned}$$

which completes the proof.

## Footnotes

1 The measures for gambling-related harms differ between the studies reviewed in this paper.

2 This ‘amount’ differs for each individual, dependent on individual characteristics.

3 Throughout the paper, ’higher’ refers to either a larger positive or a smaller negative. ’Lower’ refers to a smaller positive or greater negative.

4 This is risk aversion to the monetary aspect of gambling only. People may gain enjoyment from the excitement of the uncertainty involved in gambling, but this is captured in the psychological effects component of the utility function.

5 Professional gamblers do exist, and would have a positive expected monetary gain, and therefore may have $\alpha _{Mi} > 0$. However, these people are in the minority.

6 In Gruber and Köszegi (2001) it is given as $S_{it} = (1-d_i)(S_{i(t-1)} + A_{i(t-1)})$.

7 Note that a positive value of $U_{it}^C(A_{it},S_{it})$ implies a negative effect on total utility, and vice-versa.

8 The utility maximising gambling amount is shown in “Stage 1: Participation in Gambling” section.

9 The amount when psychological effects become net negative may be larger when cognitive biases are present, but it still exists.

10 This is by construction to simplify the model, and is not fundamental to the behaviour predicted by the model. If the amount required to satisfy cravings was greater than (10) then people would still gamble according to (10) but they would have unsatisfied cravings. Note that this would not affect how much people gamble and hence would not affect the model fundamentally. If the amount required to satisfy cravings was less than (10) then the individual would only gamble until cravings are satisfied. Since the amount they will gamble still increases as cravings increase, this also would not change the model fundamentally.

11 This is for people with $\alpha _{Pi}^O > -\alpha _{Mi}^O$. If $\alpha _{Pi}^O < -\alpha _{Mi}^O$ then the entire curve is below the x-axis. Note that if the entire curve is below the x-axis the individual may still gamble if cognitive biases cause $\alpha _{Pi}^S > -\alpha _{Mi}^S$

12 Note that if the individual has no cognitive biases this simplifies to $2\frac{\alpha _{Ci}}{d_i}>-(\alpha _{PPi} + \alpha _{MMi})$. If cognitive biases exist then $|\alpha _{PPi}^O + \alpha _{MMi}^O| > |\alpha _{PPi}^S + \alpha _{MMi}^S|$ and/ or $(\alpha _{Pi}^S + \alpha _{Mi}^S) > (\alpha _{Pi}^O + \alpha _{Mi}^O)$, both of which result in the right hand side being lower and hence increases the likelihood the inequality holds.

13 See, for example, (Young & Wohl, 2009; Ashrafioun et al., 2013; Wiehler & Peters, 2015; Thomas et al., 2009; Welte et al., 2016; Gong & Zhu, 2019; Estevez et al., 2020)

## References

[CR1] Ashrafioun L, Kostek J, Ziegelmeyer E (2013). Assessing post-cue exposure craving and its association with amount wagered in an optional betting task. Journal of Behavioral Addictions.

[CR2] Association, A. P. et al. (2013). *Diagnostic and statistical manual of mental disorders (dsm-5®)*. American Psychiatric Publication.

[CR3] Becker GS, Murphy KM (1988). A theory of rational addiction. Journal of political Economy.

[CR4] Binde P (2009). Exploring the impact of gambling advertising: An interview study of problem gamblers. International Journal of Mental Health and Addiction.

[CR5] Blaszczynski A, Nower L (2002). A pathways model of problem and pathological gambling. Addiction.

[CR6] Browne M, Bellringer M, Greer N, Kolandai-Matchett K, Langham E, Rockloff M, Abbott M (2017). Measuring the burden of gambling harm in New Zealand.

[CR7] Browne, M., Langham, E., Rawat, V., Greer, N., Li, E., Rose, J., & Goodwin, B., et al. (2016). *Assessing gambling-related harm in victoria: A public health perspective*. Victorian Responsible Gambling Foundation.

[CR8] Calado F, Griffiths MD (2016). Problem gambling worldwide: An update and systematic review of empirical research (2000–2015). Journal of Behavioral Addictions.

[CR9] Churchill SA, Farrell L (2018). The impact of gambling on depression: New evidence from England and Scotland. Economic Modelling.

[CR10] Ciccarelli M, Cosenza M, D’Olimpio F, Griffiths MD, Nigro G (2019). An experimental investigation of the role of delay discounting and craving in gambling chasing behavior. Addictive Behaviors.

[CR11] Conlisk J (1993). The utility of gambling. Journal of Risk and Uncertainty.

[CR12] Estevez A, Jáuregui P, Lopez-Gonzalez H, Mena-Moreno T, Lozano-Madrid M, Macia L (2020). The severity of gambling and gambling related cognitions as predictors of emotional regulation and coping strategies in adolescents. Journal of Gambling Studies.

[CR13] Ferris, J. A., & Wynne, H. J. (2001). *The Canadian problem gambling index*. ON: Canadian Centre on Substance Abuse Ottawa.

[CR14] Fortune EE, Goodie AS (2012). Cognitive distortions as a component and treatment focus of pathological gambling: A review. Psychology of Addictive Behaviors.

[CR15] Francis K, Dowling NA, Jackson AC, Christensen DR, Wardle H (2015). Gambling motives: Application of the reasons for gambling questionnaire in an Australian population survey. Journal of Gambling Studies.

[CR16] Gong X, Zhu R (2019). Cognitive abilities, non-cognitive skills, and gambling behaviors. Journal of Economic Behavior & Organization.

[CR17] Goodwin BC, Browne M, Rockloff M, Rose J (2017). A typical problem gambler affects six others. International Gambling Studies.

[CR18] Gruber J, Köszegi B (2001). Is addiction “rational”? Theory and evidence. The Quarterly Journal of Economics.

[CR19] Hing N, Cherney L, Blaszczynski A, Gainsbury SM, Lubman DI (2014). Do advertising and promotions for online gambling increase gambling consumption? An exploratory study. International Gambling Studies.

[CR20] Humphreys BR, Nyman JA, Ruseski JE (2021). The effect of recreational gambling on health and well-being. Eastern Economic Journal.

[CR21] Kahneman D, Sugden R (2005). Experienced utility as a standard of policy evaluation. Environmental and Resource Economics.

[CR22] Kahneman D, Wakker PP, Sarin R (1997). Back to Bentham? Explorations of experienced utility. The Quarterly Journal of Economics.

[CR23] Kapteyn A (1985). Utility and economics. De Economist.

[CR24] Levitt SD (2004). Why are gambling markets organised so differently from financial markets?. The Economic Journal.

[CR25] Livingstone C, Rintoul A (2020). Moving on from responsible gambling: A new discourse is needed to prevent and minimise harm from gambling. Public Health.

[CR26] Nyman JA, Dowd BE, Hakes JK, Winters KC, King S (2013). Work and nonpathological gambling. Journal of Gambling Studies.

[CR27] Nyman JA, Welte JW, Dowd BE (2008). Something for nothing: A model of gambling behavior. The Journal of Socio-Economics.

[CR28] Thomas AC, Allen FC, Phillips J (2009). Electronic gaming machine gambling: Measuring motivation. Journal of Gambling Studies.

[CR29] Welte JW, Barnes GM, Tidwell M-CO, Hoffman JH, Wieczorek WF (2016). The relationship between distance from gambling venues and gambling participation and problem gambling among us adults. Journal of Gambling Studies.

[CR30] Wiehler A, Peters J (2015). Reward-based decision making in pathological gambling: The roles of risk and delay. Neuroscience Research.

[CR31] Wood RT, Griffiths MD (2007). A qualitative investigation of problem gambling as an escape-based coping strategy. Psychology and Psychotherapy: Theory, Research and Practice.

[CR32] Young MM, Wohl MJ (2009). The gambling craving scale: Psychometric validation and behavioral outcomes. Psychology of Addictive Behaviors.

